# SV-HotSpot: detection and visualization of hotspots targeted by structural variants associated with gene expression

**DOI:** 10.1038/s41598-020-71168-7

**Published:** 2020-09-28

**Authors:** Abdallah M. Eteleeb, David A. Quigley, Shuang G. Zhao, Duy Pham, Rendong Yang, Scott M. Dehm, Jingqin Luo, Felix Y. Feng, Ha X. Dang, Christopher A. Maher

**Affiliations:** 1 McDonnell Genome Institute, Washington University School of Medicine, St. Louis, MO 63110 USA; 2 Department of Internal Medicine, Washington University School of Medicine, St. Louis, MO 63110 USA; 3 Department of Urology, University of California San Francisco (UCSF), San Francisco, CA 94158 USA; 4 Helen Diller Family Comprehensive Cancer Center, University of California San Francisco (UCSF), San Francisco, CA 94158 USA; 5 Department of Radiation Oncology, University of Michigan, Ann Arbor, MI 48109 USA; 6 The Hormel Institute, University of Minnesota, Austin, MN 55912 USA; 7 Masonic Cancer Center, University of Minnesota, Minneapolis, MN 55455 USA; 8 Department of Laboratory Medicine and Pathology, University of Minnesota, Minneapolis, MN 55455 USA; 9 Department of Surgery, Washington University School of Medicine, St. Louis, MO 63110 USA; 10 Siteman Cancer Center, Washington University School of Medicine, St. Louis, MO 63110 USA; 11 Department of Radiation Oncology, University of California San Francisco (UCSF), San Francisco, CA 94143 USA; 12 Department of Biomedical Engineering, Washington University in St. Louis, St. Louis, MO 63105 USA

**Keywords:** Cancer genomics, Software

## Abstract

Whole genome sequencing (WGS) has enabled the discovery of genomic structural variants (SVs), including those targeting intergenic and intronic non-coding regions that eluded previous exome focused strategies. However, the field currently lacks an automated tool that analyzes SV candidates to identify recurrent SVs and their targeted sites (hotspot regions), visualizes these genomic events within the context of various functional elements, and evaluates their potential effect on gene expression. To address this, we developed SV-HotSpot, an automated tool that integrates SV candidates, copy number alterations, gene expression, and genome annotations (e.g. gene and regulatory elements) to discover, annotate, and visualize recurrent SVs and their targeted hotspot regions that may affect gene expression. We applied SV-HotSpot to WGS and matched transcriptome data from metastatic castration resistant prostate cancer patients and rediscovered recurrent SVs targeting coding and non-coding functional elements known to promote prostate cancer progression and metastasis. SV-HotSpot provides a valuable resource to integrate SVs, gene expression, and genome annotations for discovering biologically relevant SVs altering coding and non-coding genome. SV-HotSpot is available at https://github.com/ChrisMaherLab/SV-HotSpot.

## Introduction

Structural variations (SVs) are genomic rearrangements that involve large chunks of DNA. These include deletion (loss of a genomic segment), duplication (gain multiple copies of a genomic segment), insertion (addition of a DNA sequence to the genome), inversion (one end of a genomic segment is reversed with the other end), and translocation (genomic rearrangement involving one or more chromosomes)^1^. SVs are known to contribute to phenotypic differences and various diseases including cancers^2,3^.

WGS has enabled comprehensive identification of various types of SVs targeting both the coding and non-coding tumor genome that may affect the activity or function of key driver oncogenes and tumor suppressors. This was demonstrated in a recent study of advanced prostate cancer integrating WGS, whole transcriptome, and ChIP-Seq data that showed tandem duplications involving non-coding regulatory regions are significantly associated with the expression of the androgen receptor (*AR*), a key driver of prostate cancer progression and metastasis^4,5^. However, reproducibly performing such integrative analyses on the increasing quantity of whole genome data sets is limited by the current lack of automated tools for the discovery, visualization, and interpretation of recurrent SVs and their frequent targeted sites (hotspot regions).

To address this limitation, we developed SV-HotSpot, an automated tool that integrates multiple data types including SV candidates, gene expression, copy number alterations, and genome annotations to identify, annotate, and visualize recurrent SVs and their targeted hotspot regions and assess their potential consequences on the expression of nearby genes. We applied SV-HotSpot to the whole genome and transcriptome sequencing data from 101 metastatic prostate cancer patients^4^ and rediscovered both coding and non-coding recurrent SVs known to drive prostate cancer progression.

## Results

### Hotspots of structural variations in metastatic prostate cancer

**Figure 1:**
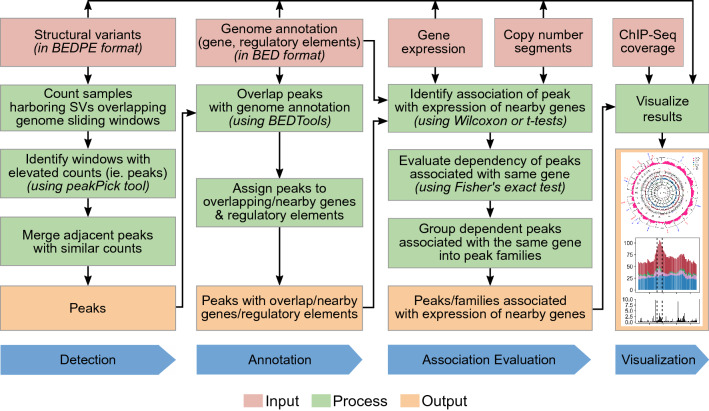
Overview of the SV-HotSpot pipeline. Recurrent (hotspot) SVs are first identified via a peak calling approach (detection), then subsequently annotated with genes and regulatory elements such as enhancers (annotation), evaluated for association with nearby gene expression (association evaluation) and visualized (visualization).

SV-HotSpot enumerates SVs targeting genomic regions and utilizes a peak calling algorithm to identify regions with elevated frequency of these events (hereby referred to as peaks or hotspots, see Methods, Fig. 1). To demonstrate that SV-HotSpot is able to detect biologically relevant recurrent SVs, we applied it to WGS and matched RNA-Seq data from 101 metastatic prostate cancer patients^4^. To identify peaks corresponding to regulatory regions, we additionally included annotated enhancers^6^ and H3K27ac ChIP-Seq (Chromatin Immunoprecipitation Sequencing) data from prostate cancer patients^7^.

**Figure 2:**
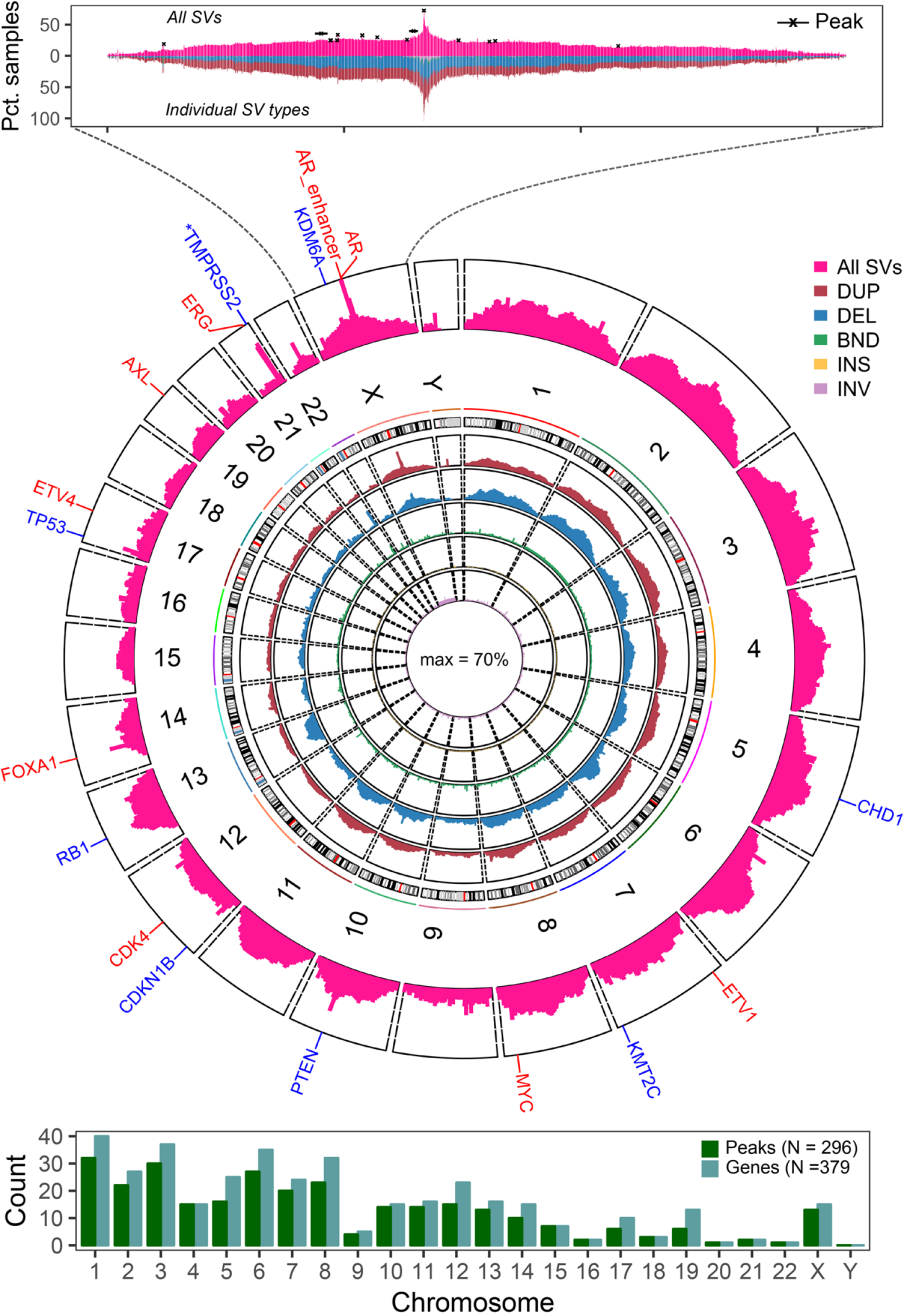
SV-HotSpot identifies hotspots of recurrent SVs associated with genes well characterized in metastatic prostate cancer. The circos plot (top panel) shows the percentage of samples harboring any type of SVs (outer track) or individual types of SVs (inner tracks) targeting genomic windows. Genes known in prostate cancer and found to have altered expression in the presence of SVs targeting hotspots are annotated in the outer gene track (red—up-regulated in presence of SVs, blue—down-reguated in presence of SVs, *—3′ truncated in fusion leading to lower gene expression estimate). The bottom panel shows numbers of SV hotspots and genes associated with these hotspots for individual chromosomes.

In total, we identified 296 SV hotspot sites associated with altered expression of 379 nearby genes (Fig. 2, Supplementary Tables S1, S2). SV-HotSpot identified and highly ranked hotspot sites harboring SVs associated with expression of many genes known to drive prostate cancer progression, metastasis, and treatment resistance (Fig. 2, Supplementary Table S1). Interestingly, various SV types were found to be recurrent and associated with altered expression of tumor suppressors and oncogenes including tandem duplication, chromosomal translocation, and copy number alteration.

### Hotspots of tandem duplications targeting genes and regulatory elements

**Figure 3:**
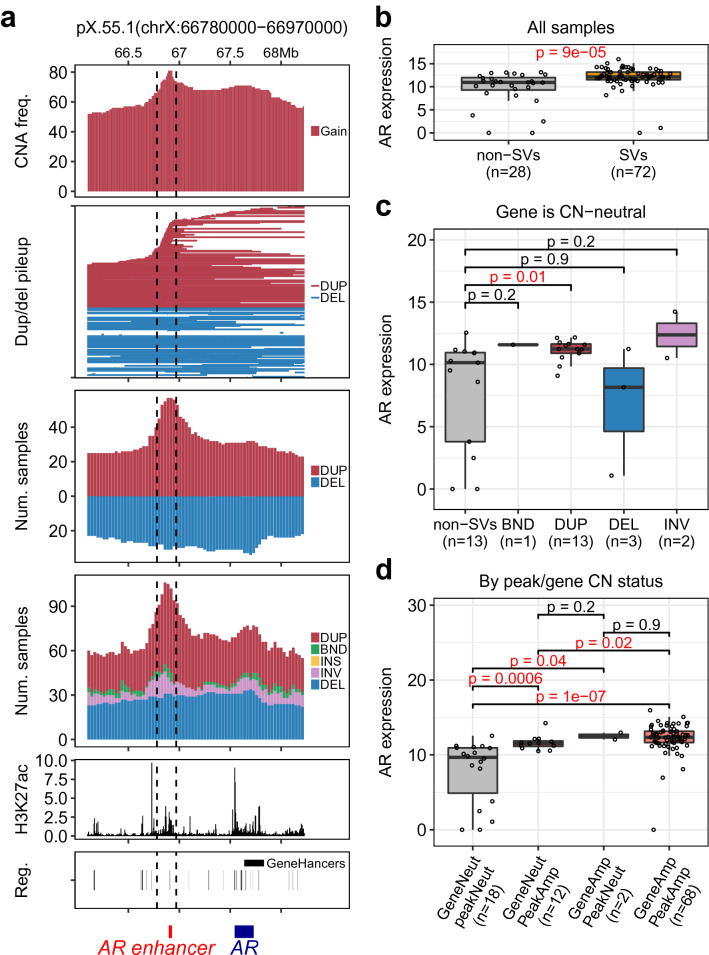
SV-HotSpot detects and visualizes recurrent SVs targeting the non-coding *AR* enhancer and is associating with increased *AR* expression. (**a**) Visualization of recurrent SVs targeting the peak (bounded by vertical dashed lines). From top to bottom are aggregation of copy number alterations, pileup of duplication/deletion regions, aggregation of duplication/deletion regions, aggregation of all types of SVs, H3K27ac histone modification ChiP-Seq coverage, and enhancer annotation from Genehancer database. DUP—duplication, DEL—deletion, INS—insertion, INV—inversion, and BND—translocation (**b**) Comparison of *AR* expression between samples with SVs targeting the peak and those lacking SVs targeting *AR* enhancer peak. (**c**) Comparison of *AR* expression between samples with different types of SVs targeting the peak. (**d**) Comparison of *AR* expression between samples grouped by the presence/absence of copy number targeting *AR* gene body and the peak. The whole figure is the direct output of SV-Hotspot visualization.

Recent studies highlighted the critical roles of tandem duplications in cancers including prostate cancer^4,5,8,9^. SV-HotSpot identified various hotspots of tandem duplications targeting both coding and non-coding regions in metastatic prostate cancer. Most notably, SV-HotSpot detected a peak of recurrent SVs primarily comprised of tandem duplications targeting a non-coding enhancer located at ~ 625 kb upstream of *AR*, a key driver of prostate cancer progression, treatment resistance, and metastasis^10^ (Fig. 3). This region was also found to be amplified in 81% of patients (Fig. 3a, top track). SV-HotSpot detected a strong association between the presence of tandem duplications or copy number gain in this region with increased *AR* expression (Fig. 3b–d). Moreover, SV-HotSpot annotated this region with an active enhancer and an enriched H3K27ac occupancy (Fig. 3a, bottom two tracks). These results are consistent with the recent discovery of an *AR* enhancer that regulates *AR* gene expression and is highly frequently duplicated in prostate cancer metastasis^4,5,11^.

**Figure 4:**
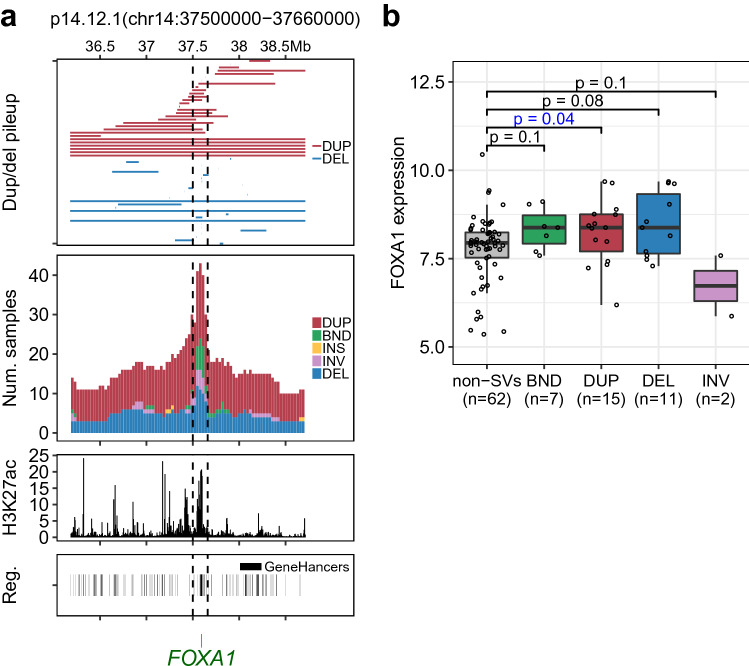
SV-HotSpot detects recurrent SVs targeting *FOXA1* locus. (**a**) Visualization of *FOXA1* locus. From top to bottom are pileup of duplication/deletion, aggregation of sample counts for different SV types, H3K27ac ChiP-Seq coverage from primary prostate cancer patients, enhancer annotation from GeneHancers. (**b**) Comparison of *FOXA1* expression between samples harboring SVs and those do not (restricted to samples in which *FOXA1* is copy neutral).

In addition to the rediscovery of the *AR* enhancer, SV-HotSpot also detected peaks of frequent tandem duplications targeting both the coding and non-coding regions of *MYC* and *FOXA1* loci (Fig. 4, Supplementary Tables S1, S2). Interestingly, SV-HotSpot reported an association of tandem duplication targeting both coding and non-coding regions of *FOXA1* locus with increased gene expression (Fig. 4), consistent with a recent report^12^. *MYC* was found to be overexpressed in the presence of copy number gain in its hotspot locus. There was also an enrichment of tandem duplication targeting both coding and non-coding regions near *MYC* (Supplementary Table S2).

### Hotspots of deletions and translocations associated with ETS gene fusions

**Figure 5:**
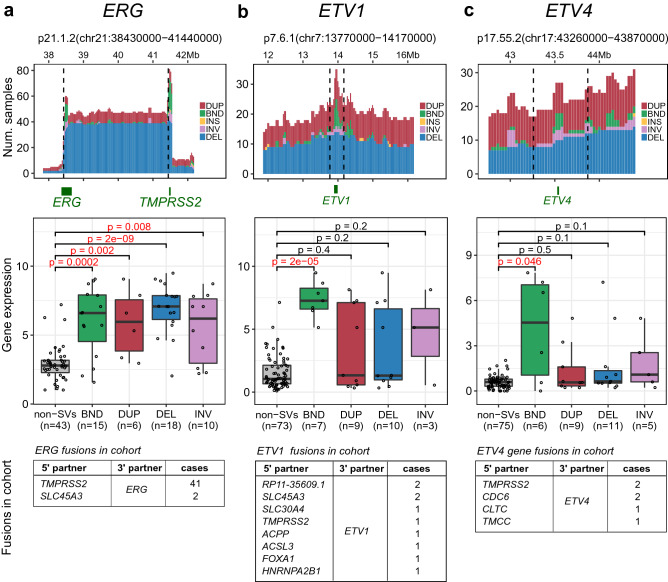
SV-HotSpot detects recurrent deletions and translocations corresponding to the ETS family gene fusions. From left to right are results for *ERG *(**a**),* ETV1* (**b**) *and ETV4* (**c**)*.* In each panel, from top to bottom are (1) stack histogram of sample counts for different types of SVs within the peak (bounded by black dashed lines), (2) comparison of expression of the gene between samples harboring individual SV types and those without any SVs (restricted to samples whose gene is copy neutral), and (3) list of gene fusions in cohort whose 3′ partner is the gene (not part of SV-HotSpot visualization). *ERG* hotspot is enriched for deletions corresponding to *TMPRSS2-ERG* fusions and translocations including those that create*—ERG* fusions with different 5′ partners, all associated with increased *ERG* expression. *ETV1* and *ETV4* peaks were enriched for translocations corresponding to various fusions and associated with increased gene expression. Note that we used expression of *ERG*,* ETV1*, and *ETV4* from the original publication that was normalized using the full length genes, and thus could be lower than the true fusion expression. This does not change our conclusion as the actual effect of translocations on fusion expression could be higher.

ETS gene fusions are well characterized somatic genome rearrangements that drive prostate cancer tumorigenesis and defines a distinct molecular subtype^13–15^. SV-HotSpot identified and highly ranked hotspots that harbor deletions or chromosomal translocations resulting in gene fusions and increased expression of ETS transcription factor family genes. For instance, SV-HotSpot reported the highest ranked peak in chromosome 21 consisting primarily of recurrent deletion events that targeted the genomic region between *TMPRSS2* and *ERG* genes corresponding to the *TMPRSS2-ERG* fusion (Fig. 5a). Deletion of the region between *TMPRSS2* and *ERG* was found to be strongly associated with increased *ERG* expression (Fig. 5a). There was also an enrichment of translocations at *ERG* locus that were found to be associated with increased *ERG* expression. These translocations included events corresponding to *ERG* gene fusions with different 5′ partner genes (Fig. 5a).

Additionally, SV-HotSpot identified hotspots targeting other ETS genes including *ETV1* and *ETV4* (Fig. 5b,c, Supplementary Table S1). Interestingly, while many types of SVs were observed in *ETV1* and *ETV4* loci, only chromosomal translocations were found to be associated with increased expression of these genes. These enriched translocations corresponded to the inter-chromosomal rearrangements that created *ETV1* gene fusions (10 patients, 10%, Fig. 5b) and *ETV4* gene fusions (6 cases, 6%, Fig. 5c).

### Hotspot of structural variations disrupting tumor suppressors

**Figure 6:**
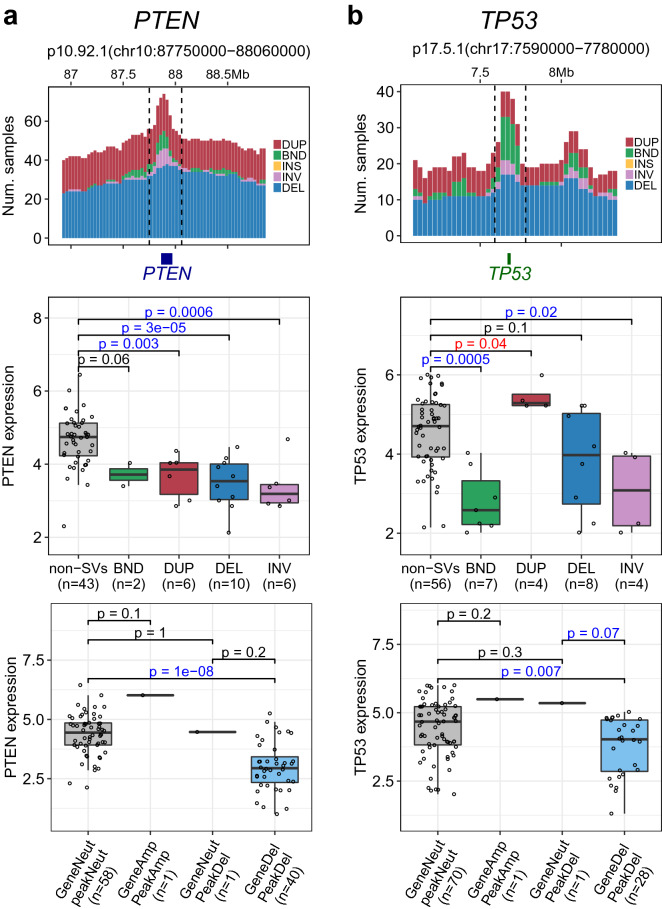
SV-HotSpot identifies hotspots of SVs disrupting tumor suppressor genes *PTEN* and *TP53*. (**a**) Hotspot of SVs targeting *PTEN* locus. From top to bottom are stack histogram of sample counts for different SV types within the peak region (bounded by dashed black lines), comparison of *PTEN* expression between SV types in patients whose *PTEN* is copy neutral, and comparison of *PTEN* expression between samples whose peak/gene are copy neutral/loss. (**b**) Hotspot of SVs targeting *TP53* locus. As shown, *PTEN* expression is significantly decreased in the presence of deletion, duplication, translocation, or inversion while *TP53* expression is significantly decreased in the presence of translocation or inversion. Both genes show significantly decreased expression when affected by copy number loss.

Tumor suppressor genes are often inactivated in cancers by various mechanisms including structural rearrangements^4,16,17^. SV-HotSpot detected hotspot sites associated with decreased expression of well-characterized tumor suppressor genes including *PTEN*, *TP53*, *RB1,* and *CDKN1B* (Fig. 6, Supplementary Table S1). It is notable that various SV types were found to target *PTEN* and *TP53* loci and regardless of the SV types, events targeting these tumor suppressors often found to be associated with decreased gene expression (Fig. 6). For example, all types of SVs targeting *PTEN* hotspot were associated with decreased *PTEN* expression including deletion, duplication, translocation, and inversion (Fig. 6a, Supplementary Table S1). This is consistent with previous report that *PTEN* is often disrupted by various forms of chromosomal rearrangements^4,16^. Similarly, *TP53* expression was decreased in the presence of deletion, translocation, and inversion (Fig. 6b, Supplementary Table S1).

Taken together, via reanalysis of public datasets, we showed that SV-HotSpot was able to detect hotspots of recurrent SVs known to contribute to prostate cancer development, progression, metastasis, and treatment resistance.

## Discussion

Here, we present SV-HotSpot, an automated pipeline to identify, annotate, and visualize hotspots of recurrent SVs and evaluate their potential consequences on the expression of nearby genes. Despite the great success of recent studies in identifying recurrent SVs and assessing their impact^4,18^, these approaches require significant amount of work and ad-hoc analyses to integrate multiple types of data and evaluate the potential effect of SVs on gene expression. SV-HotSpot seamlessly integrates and analyzes multiple data types including SV candidates, gene expression, copy number alterations, and functional elements to discover recurrent SV hotspots. Additionally, it comprehensively evaluates the associations between recurrent SVs and various genome annotation/functional elements and potential consequence on gene expression. Furthermore, SV-HotSpot provides useful visualizations to facilitate the interpretation of the results. As a fully automated tool, SV-HotSpot allows for customized and reproducible analyses.

SV-HotSpot uses a sliding window approach that is a generalization of the frequently used genomic binning approach and allows smoothing of the sample counts for effective peak calling. The use of peak calling algorithm to identify recurrent SVs in SV-HotSpot enables systematic identification of regions with statistically elevated frequency of SVs that are more likely functional. This approach is similar to those employed by GISTIC tool to identify genes recurrently targeted by copy number alteration^19^. Compared with GISTIC which focuses on identification of focal copy number alteration, SV-HotSpot additionally integrates a broad spectrum of structural variations, gene expression, and regulatory elements, and thus was able identify other types of recurrent SVs targeting regulatory elements driving gene expression such as tandem duplication of the *AR* enhancer. Our approach is also complementary to existing network biology approaches such as those utilizing molecular interaction data and information flow method to find association between genes and diseases^20^.

Through our reanalysis of metastatic prostate cancer patient data, we demonstrated the utility of SV-HotSpot for detecting biologically relevant and well-characterized recurrent SVs that regulate the expression of nearby genes. We identified key prostate cancer driver genes as the most significantly associated genes with their commonly known recurrent SVs including tandem duplication of the *AR* enhancer, deletion of the *TMPRSS2-ERG* region, and genomic disruption of *PTEN*. Moreover, our thorough evaluation of expression association allowed us to identify specific types of SVs known to affect gene expression including those with lower frequency such as translocations resulting in gene fusions of *ETV1* and *ETV4*, and tandem duplication affecting *FOXA1*. Overall, SV-HotSpot is a valuable tool for the cancer research community to integrate the growing whole genome, transcriptome, and epigenetic data to discover biologically relevant SV hotspots. Although the tool was applied to human cancer data in this study, it can also be applied to data from other species and diseases.

## Methods

SV-HotSpot consists of four main steps (Fig. 1): (1) detection of SV hotspots, (2) annotation of SV hotspots, (3) evaluation of the association of hotspot SVs on expression of nearby genes, and (4) visualization.

In the first step, SV-HotSpot identifies regions with elevated frequency of SVs by utilizing a peak calling approach on counts of samples harboring SVs targeting sliding windows over each chromosome. First, it uses the SV candidates (in BEDPE, Browser Extensible Data Paired-End format) as an input and counts the number of samples harboring SV breakpoints (in the case of translocations, insertions, and inversions) or regions (in the case of duplications and deletions) overlapping with sliding windows. The entire duplication/deletion regions were considered because these events directly affect the contained genome elements by changing their copy numbers while other events only potentially affect elements near their break ends. SV-HotSpot then applies the peakPick peak calling algorithm^21^ to identify windows (referred to as ‘peaks’ thereafter) where counts are significantly higher than those of the surrounding windows. Peaks occurring in at least a certain percentage of SV samples (defined by users) are identified as potential peaks. Once all potential peaks are identified, SV-HotSpot applies a peak merging algorithm to group adjacent peaks with similar sample counts, as those are likely resulted from the same genome rearrangements that target the same sites. The peak merging algorithm works by first identifying clusters of adjacent peaks where any two contiguous peaks are within a predefined distance. Next, it selects the top peak (peak with the highest sample count) among a peak cluster and moves upstream and downstream to merge peaks until it observes *k* peaks (*k* is small, e.g. 1–3, predefined) with significant change of sample counts compared with the top peak (predefined parameter *delta*, e.g. 5%). This process is repeated until no peaks in the cluster remain. The merged peaks are then considered final peaks for subsequent analyses.

In the second step, identified peaks are annotated with nearby genes and overlapping regulatory elements such as enhancers and promoters, provided as input in BED (Browser Extensible Data) format using BEDTools^22^. All annotated peaks are then summarized and output in BED format.

In the third step, gene expression and copy number data are incorporated to evaluate if the presence of SVs at each peak is associated with altered expression of each nearby gene. A *Wilcoxon rank-sum* test or *t-test* (chosen by users) is used to compare the expression of the nearby gene between samples harboring and not harboring a hotspot SV. The same test is also performed within sample stratifications based on copy number status (gain, loss, or neutral) of the nearby gene. More specifically, SV-HotSpot applies 12 different comparisons (illustrated below) in order to determine the association between a hotspot and a nearby gene.

1. Comparison of the expression of a nearby gene between samples harboring hotspot SVs and those not harboring hotspot SVs without considering copy number status of the gene to determine the overall association between recurrent SVs and the expression of the gene.
2. Similar to (1), five comparisons are also performed between samples harboring each of the five individual SV types (duplication, deletion, translocation, insertion, and inversion) targeting the hotspot and those without any SVs targeting the hotspot to identify whether the overall expression association is derived by specific SV types.
3. Comparison of the expression of a nearby gene between samples harboring hotspot SVs and those not harboring hotspot SVs but only among samples without copy number alteration of the gene to determine whether the association is derived by SVs without the confounding impact of copy number alterations of the gene.
4. Similar to (3), five comparisons are also performed between samples harboring each of the five individual SV types (duplication, deletion, translocation, insertion, and inversion) targeting the hotspot and those without any SVs targeting the hotspot to identify whether the expression association is derived by specific SV types.

We determine that the presence of SVs at a peak is associated with the expression of a nearby gene if any of the above comparisons results in the rejection of the null hypothesis that there is no difference in gene expression between groups. To achieve this, the Fisher’s method^23^ is used to combine the *p*-values of these tests. Subsequently, the false discovery rate (FDR) was estimated using the Benjamini–Hochberg^24^ using the Fisher’s combined p-values.

Additionally, SV-HotSpot groups dependent peaks that are likely driven by the same SV events and have similar consequences on the expression of nearby genes into peak families. To achieve this, after the peaks are identified, those associated with the same gene are tested for dependency using a Fisher’s exact test. If peaks are found to be dependent (significant overlap of the samples harboring SVs between the peaks), they are grouped as a peak family. The top-ranked peak (the peak with highest count of samples harboring the hotspot SVs) is reported as the representative of the peak family.

Finally, SV-HotSpot generates multiple visualizations (Fig. 2 top panel, Fig. 3) for interpretation of the genomic context and the association between SVs and gene expression. In these visualizations, SV-HotSpot overlays multiple tracks to show copy number alterations, SV breakpoint aggregation, segments of duplications and deletions, gene and regulatory element annotation, and ChIP-Seq coverage in close proximity to the peaks and its nearby genes (Fig. 3a). In addition, the expression of nearby genes is plotted to highlight associations with recurrent SVs (Fig. 3b), with different types of SVs (Fig. 3c), and with copy number status of both the peak and nearby genes (Fig. 3d). SV-HotSpot also provides an additional visualization of the distribution of identified peaks on each chromosome (Fig. 2, top panel). Furthermore, SV-HotSpot generates a custom track file for each chromosome that can be viewed on the UCSC Genome Browser.

For analyses reported in the Results section, SV-HotSpot was run using a sliding window size of 100 kb, step size of 30 kb, peak merging distance of 50 kb, default parameters for peakPick, and peak merging parameters *k* = 1, *delta* = 5%. Only peaks smaller than 500 kb (except for those associated with altered expression of a COSMIC census gene^25^) and present in at least 15% of samples with an FDR < 0.05 and at least one of 12 expression associations significant at a *p-value* < 0.05 (Wilcoxon test) were retained. Additionally, only genes with mean expression > 10 TPM (Transcripts Per Million) in a group from a significant comparison were retained.

## Supplementary information

Supplementary Tables.

## Data Availability

SV-HotSpot is a Linux-based command-line pipeline implemented in R and Perl and can be run as a Docker container or Bioconda package. SV-HotSpot is available at https://github.com/ChrisMaherLab/SV-HotSpot.
